# Self-reported eating rate is associated with weight status in a Dutch population: a validation study and a cross-sectional study

**DOI:** 10.1186/s12966-017-0580-1

**Published:** 2017-09-08

**Authors:** Janet H.W. van den Boer, Jentina Kranendonk, Anne van de Wiel, Edith J.M. Feskens, Anouk Geelen, Monica Mars

**Affiliations:** Division of Human Nutrition, Wageningen University, P.O. Box 17, 6700 AA Wageningen, The Netherlands

**Keywords:** Eating behavior, Eating style, Microstructure of eating, Eating fast, Speed of eating, Overweight

## Abstract

**Background:**

Observational studies performed in Asian populations suggest that eating rate is related to BMI. This paper investigates the association between self-reported eating rate (SRER) and body mass index (BMI) in a Dutch population, after having validated SRER against actual eating rate.

**Methods:**

Two studies were performed; a validation and a cross-sectional study. In the validation study SRER (i.e., ‘slow’, ‘average’, or ‘fast’) was obtained from 57 participants (men/women = 16/41, age: mean ± SD = 22.6 ± 2.8 yrs., BMI: mean ± SD = 22.1 ± 2.8 kg/m^2^) and in these participants actual eating rate was measured for three food products. Using analysis of variance the association between SRER and actual eating rate was studied. The association between SRER and BMI was investigated in cross-sectional data from the NQplus cohort (i.e., 1473 Dutch adults; men/wome*n* = 741/732, age: mean ± SD = 54.6 ± 11.7 yrs., BMI: mean ± SD = 25.9 ± 4.0 kg/m^2^) using (multiple) linear regression analysis.

**Results:**

In the validation study actual eating rate increased proportionally with SRER (for all three food products *P* < 0.01). In the cross-sectional study SRER was positively associated with BMI in both men and women (*P* = 0.03 and *P* < 0.001, respectively). Self-reported fast-eating women had a 1.13 kg/m^2^ (95% CI 0.43, 1.84) higher BMI compared to average-speed-eating women, after adjusting for confounders. This was not the case in men; self-reported fast-eating men had a 0.29 kg/m^2^ (95% CI -0.22, 0.80) higher BMI compared to average-speed-eating men, after adjusting for confounders.

**Conclusions:**

These studies show that self-reported eating rate reflects actual eating rate on a group-level, and that a high self-reported eating rate is associated with a higher BMI in this Dutch population.

## Background

Eating rate, the amount of food consumed per unit of time, has attracted attention for its potential role in preventing and treating obesity [1]. Slower eating is expected to reduce food intake and consequently body weight. Calories that pass quickly through the oral cavity go largely undetected and do not bring about an adequate satiety response, resulting in an increased intake [2]. Moreover, eating rate is a personal characteristic – some people tend to eat faster than others, or vice versa [3–5] – and eating rate could therefore affect long-term energy intake and consequently body weight. A recent meta-analysis has shown that the amount of food eaten can be altered by (experimentally) manipulating eating rate [6]. Furthermore, research indicates that eating rate might affect long-term energy intake and weight status [1, 7–9].

The relation between (self-reported) eating rate, energy intake and BMI has been studied in a number of cross-sectional studies, predominantly Asian. The results in general indicate that a higher self-reported eating rate (SRER) is associated with a higher long-term energy intake, though the results are not conclusive [10, 11]. Furthermore, a recent study by Fogel et al. [12] showed that the actual eating rate of Singaporean children was positively associated with BMI. Regarding adults, a recent review and meta-analysis by Ohkuma et al. [7] showed that self-reported fast eaters were more likely to be overweight (BMI ≥ 25 kg/m^2^) compared to self-reported slow eaters; All studies reported a positive association between eating rate and weight status, although there was a large variation in magnitude of the association. In addition, positive associations have been found between SRER and weight gain in longitudinal studies [13, 14]. For example, Tanihara et al. [13] found that male office workers who reported to be fast eaters on average gained 1.9 kg over a period of 8 years while the other male office workers only gained 0.7 kg on average.

This research consistently showed that SRER is associated with energy intake and BMI, but the generalizability of these findings is questionable. The studies in this field of research are limited to Asian populations, predominantly Japanese. To date only Leong et al. [15] investigated the association between SRER and BMI in a non-Asian population (i.e., New Zealand). This study, however, only included women, relied on self-reported data for height and weight, and did not collect data on energy intake. More research is needed to see if similar (positive) associations between SRER, energy intake and BMI exist in non-Asian populations, despite differences in diet, habits and ethnicity [7].

Furthermore, data on the validity of self-reported eating rate is limited [10, 16–18]. To our knowledge only Petty et al. [18] validated SRER against actual eating rate (g/min). They showed that, on a group-level, actual eating rate increased with increasing SRER-categories (i.e., slow, medium and fast). They, however, only validated SRER for one food product (i.e., pasta), and they did not address how well SRER reflects the actual eating rate of individuals.

Hence we aimed to validate self-reported eating rate and investigate its relation with energy intake and objectively assessed weight status in Dutch men and women. First we conducted a laboratory study validating self-reported eating rate in three foods varying in structure, after which we analyzed self-reported eating rate and different measures of weight status (i.e., BMI, waist circumference and body fat percentage) in a large, Dutch cohort study (i.e., NQplus [19]).

## Methods

### Study 1: Validation SRER

#### Study population and design

Students were recruited through posters at university buildings and student housing in Wageningen. Students who did not like the food products offered were excluded from participation. In total 64 students participated. Seven of them were excluded from the analyses; six because of a technical error and one because of mobile phone usage during the test session.

SRER and actual eating rate were obtained from participants during a single visit to the university in October–November 2013. This allowed for comparison between SRER and actual eating rate within persons. Participants were told that the aim of the study was to pilot test lunch products for another study.

#### Procedure

Participants were instructed not to eat anything in the 2 h prior to their lunch at the university. First they filled out a questionnaire on their eating behavior which included a question on eating rate: “How would you describe your eating rate compared with others? ‘Very slow’, ‘slow’, ‘average’, ‘fast’ or ‘very fast’?”. This question was based on previous research [10, 15]. Subsequently the participants received three lunch products: first a soft bun with cheese, then apple, and finally vanilla custard. Serving sizes differed between participants. The total lunch offered represented a normal lunch in terms of energy content; i.e., 20% of the daily energy requirement of the individual participants, which was estimated using the Schofield equation while assuming a moderate physical activity level [20, 21]. Participants pressed the spacebar of the laptop in front of them with the first bite of a product and again when they swallowed the last bite. The time between pressing the spacebars was recorded, which represents the time spent eating. Intake was measured by weighing the products prior to and after consumption. Actual eating rate was determined by dividing the intake in grams by the time spent eating in minutes for each product separately.

Furthermore, before the consumption of each product and at the end participants rated their level of satiety using visual analogue scales (0-100 mm); Feelings of hunger (Not at all-Extremely), fullness (Not at all-Extremely), satiety (Not at all-Extremely), desire to eat (Very weak-Very strong) and prospective consumption (Nothing at all-A very large amount) were rated [22]. Overall satiety scores were calculated by extracting the average of the scores for hunger, desire to eat and prospective consumption from the average of the scores for fullness and satiation [22]. Moreover, after the consumption of each product participants indicated how much they liked the product (1, Dislike very much – 5 Like very much).

### Study 2: Association between SRER and weight status

#### Study population and design

This study investigates data from NQplus, an ongoing cohort study designed to: validate a newly developed FFQ, start a reference database for nutrition research and study associations between diet and intermediate health outcomes [19, 23]. The cohort consists of adults (20–70 years old) randomly selected from households in Wageningen, Renkum, Ede, Arnhem and Veenendaal. Participants were recruited via letters and emails between May 2011 and March 2013. In total 2048 people were included. SRER was available of 1642 participants; The other participants either did not answer the eating rate question, or dropped out before receiving the question. Finally, 1473 participants were included in the analyses, as other data (i.e., data on age, smoking, education level, emotional eating, restraint eating, and/or external eating) was missing for 169 out of the 1642 participants with SRER.

Since registration the participants received a number of questionnaires (which twice included the eating rate question). Additionally, anthropometric measurements were taken. The collected data was used for the cross-sectional analysis of the association between SRER and weight status.

#### Online questionnaires

##### General characteristics

At baseline participants reported their highest completed education, which was categorized into three groups; low (i.e., no education, primary education, lower or preparatory vocational education, or lower general secondary education), medium (i.e., intermediate vocational education or apprenticeship, or higher general secondary education or pre-university secondary education) and high (i.e., higher vocational education or university). Additionally, the participants completed a semi-quantitative food frequency questionnaire (FFQ) on last month’s intake which was used to calculate average daily energy intake. This FFQ has been found to be valid to assess mean energy intake in large samples and for ranking individuals [24].

Furthermore, the participants received the ‘Dutch Eating Behavior Questionnaire’ (DEBQ) [25]. The DEBQ contains 33 items; 13 items reflect emotional eating, ten items reflect external eating and ten items reflect restrained eating. Average scores were calculated to obtain sub scores for emotional, external and restrained eating. Usual physical activity was assessed using two questionnaires: i.e., the ‘Activity Questionnaire for Adults and Adolescents’ (AQUAA) for sedentary activity [26], and the ‘Short questionnaire to assess health-enhancing physical activity’ (SQUASH) for moderate-to-vigorous activity [27]. Both sedentary and moderate-to-vigorous activity were determined in minutes per week, and were converted to hours per week for the analyses.

##### Self-reported eating rate

Participants twice received a questionnaire that included the eating rate question (see validation study). The median time in between was 12.1 months. In the analyses we used the SRER off the first time the participants answered the eating rate-question. SRER-data from the second time the participants answered the eating rate-question was only used to test repeatability.

#### Anthropometrics

Anthropometric measurements were performed twice. The median time in between was 12.9 months. Height was measured, without footwear, to the nearest 0.1 cm using a stadiometer (SECA, Hamburg, Germany). Weight was measured to the nearest 0.1 kg using a digital scale (SECA, Hamburg, Germany), after taking of footwear and heavy clothes and removing heavy items from the pockets. Waist circumference was measured between the lowest rib and the iliac crest to the nearest 0.5 cm using a non-elastic flexible tape (SECA, Hamburg, Germany), after removing thick clothes from that area. This measurement was performed twice and the average of those measurements was used for analyses. Finally, body fat percentage was measured using a DEXA-scan (Lunar Prodigy Advance; GE Healthcare, Madison, Wisconsin, United States).

For the current analyses we used the anthropometric data of the visit closest to the first time the eating rate-question was answered. On average there were 165 (±91) days between answering the eating rate-question for the first time and the anthropometric measurements.

### Statistical analyses

SPSS (IBM SPSS Statistics, Version 20, IBM Corporation, Armonk, NY, USA) was used for the statistical analyses. Means and standard deviations are given, unless stated otherwise. *P*-values of <0.05 were considered statistically significant. Normality was judged by visual inspection using QQplots; all data were normally distributed.

SRER was split into three categories for the analyses, as in both studies only few participants reported to be a very slow or very fast eater; The ‘very slow’ category was combined with the ‘slow’-category and the ‘fast’ category with the ‘very fast’-category.

#### Study 1: Validation SRER

By means of analysis of variance it was investigated whether there was a linear trend between SRER and actual eating rate. Post-hoc analyses (Gabriel’s procedure) were performed to identify differences in actual eating rate between SRER-categories. To investigate how well SRER reflects the actual eating rate of individuals, the level of agreement between SRER and tertiles of actual eating rate was determined by calculating kappa (ĸ) (i.e., chance-corrected proportional agreement) [28].

Furthermore, correlation analyses were performed to investigate the association between the eating rate of the lunch products, between eating rate and liking, and between eating rate and satiety. Independent t-tests were performed to investigate whether eating rate differed between men and women.

#### Study 2: Association between SRER and weight status

Data were analyzed for the total population and for men and women separately, as both eating rate and BMI are sex-dependent [29, 30]. Kappa (ĸ) was calculated to assess the level of agreement between answers of participants that answered the eating rate-question on two separate occasions [28]. One-way analyses of variance, independent samples T-tests and chi-square tests were performed to check whether the participant characteristics (e.g., weight status and intake) differed between males and females and between the SRER-categories. Linear regression analysis was performed to investigate whether there was a linear trend between the SRER-categories and participant characteristics.

Furthermore, multiple linear regression analyses were performed to investigate the association between SRER and BMI with adjustment for potential confounders. First a crude model was tested with two dummy variables of SRER; one for comparing fast with average eating rate, one for comparing slow with average eating rate. In a second model age, smoking and education level were added. In the third and main model DEBQ-scores (i.e., emotional, restrained and external eating) were added. In an additional model, ‘Model X’, energy intake, moderate-to-vigorous activity and sedentary activity were added to the main model. Suspected under reporters of energy intake (i.e., reported energy intake/calculated basal metabolic rate < 1.35 [31]) were excluded. Due to the exclusion of suspected under reporters and missing data ‘Model X’ is based on a small subset of the total sample, and is therefore not considered to be the main model. Furthermore, it was investigated whether there was a linear trend between the SRER-categories and BMI in the different models by replacing the dummy variables with the categorical variable for SRER.

Finally, odds ratios for overweight (i.e., BMI ≥ 25 kg/m^2^) were determined for self-reported fast eaters (compared to self-reported slow plus average-speed eaters) by means of logistic regression analyses, taking into account potential confounders (i.e., age, smoking, education level, emotional eating, restrained eating and external eating).

## Results

### Study 1: Validation SRER

**Fig. 1:**
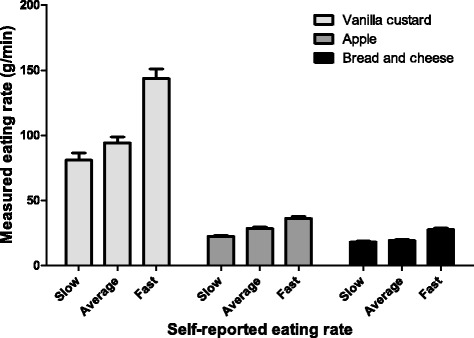
Actual eating rate within self-reported eating rate-categories (*n* = 57)

In total 57 (men/wome*n* = 16/41) participants (22.6 ± 2.8 years old, self-reported BMI of 22.1 ± 2.8 kg/m^2^) were included. Eleven participants reported to be a slow eater (i.e., very slow (*n* = 1) or slow (*n* = 10)), 27 participants reported to be an average speed eater, and 19 participants reported to be a fast eater (i.e., fast (*n* = 18) or very fast (*n* = 1)). Eating rate (g/min) increased proportionally with SRER for all three lunch products (bread with cheese *F* (1, 51) = 10.45, *P* < 0.01; apple *F* (1, 43) = 12.79, *P* < 0.01; vanilla custard *F* (1, 49) = 13.12, *P* < 0.01) (Fig. 1). Post-hoc analyses showed that eating rate was significantly higher in self-reported fast eaters compared to self-reported slow and average-speed eaters, but did not differ between self-reported slow and average-speed eaters.

The level of agreement between SRER and actual eating rate-tertiles was fair; for all three lunch products a ĸ-value of 0.25 was found [28]. Within all lunch products actual eating rate-tertiles corresponded with SRER in 50% of the cases, while in about 10% of the cases the actual eating rate-quartiles and SRER showed the opposite; e.g., indicated to be a slow eater, while actual eating rate was in the highest tertile.

Furthermore, how fast participants consumed one lunch product was correlated with how fast they ate the other lunch products (bread with cheese x apple, *r* = .54 *P* < 0.001; bread with cheese x vanilla custard, *r* = .50 *P* < 0.001; apple x vanilla custard, *r* = .69 *P* < 0.001). Liking was correlated with eating rate in vanilla custard (*r* = .37, *P* < 0.01), but not in bread with cheese (*r* = −.02, *P* = 0.90) and apple (*r* = .16, *P* = .28). Moreover, eating rate was not associated with the satiety score at the start of consumption for all three lunch products (bread with cheese, *r* = −.13 *P* = 0.37; apple, *r* = .07 *P* = 0.66; vanilla custard, *r* = −.12 *P* = 0.41). Finally, men ate all three lunch products faster than women (bread with cheese *t* (52) = −4.84, *P* < 0.001; apple *t* (44) = −6.22, *P* < 0.001, vanilla custard *t* (50) = −4.65, *P* < 0.001).

### Study 2: Association between SRER and weight status

Data from 741 men and 732 women is included in the main analyses (Table 1). On average, men were 57.5 ± 10.6 years old and had a BMI of 26.4 ± 3.5 kg/m^2^ whereas women were 51.8 ± 12.0 years old and had a BMI of 25.3 ± 4.4 kg/m^2^. Collectively, ages ranged from 21.7–77.0 yrs. old and BMI from 16.8–57.6 kg/m^2^ for the two groups.

**Table 1:** Characteristics of the men and women participating in NQplus

	Men			Women			*P*
	n	Mean	SD	n	Mean	SD	
Age (yrs.)	741	57.5	10.6	732	51.8	12.0	<0.001^a^
Height (cm)	741	180.4	6.8	732	168.6	6.3	<0.001^a^
Weight (kg)	741	86.0	12.8	732	71.8	13.2	<0.001^a^
BMI (kg/m^2^)	741	26.4	3.5	732	25.3	4.4	<0.001^a^
Waist circumference (cm)	737	96.9	10.7	731	85.8	11.6	<0.001^a^
Body fat percentage (%)	683	24.7	6.6	613	34.9	7.7	<0.001^a^
Energy intake (MJ/day)	691	9.5	2.6	654	7.8	2.1	<0.001^a^
Energy intake (MJ/day)^c^	288	11.7	2.1	296	9.4	1.6	<0.001^a^
Emotional eating score	741	1.94	0.65	732	2.28	0.74	<0.001^a^
Restrained eating score	741	2.87	0.73	732	3.13	0.68	<0.001^a^
External eating score	741	2.70	0.43	732	2.71	0.45	0.48^a^
Moderate-to-vigorous activity (hours/week)	696	33.9	18.5	666	35.3	16.9	0.13^a^
Sedentary activity (hours/week)	696	36.6	21.0	666	36.4	34.0	0.90^a^
		n	%		n	%	
Prevalence of overweight^d^		460	62.1		322	44.0	<0.001^b^
Prevalence of obesity^e^		118	15.9		91	12.4	0.06^b^
Self-reported eating rate							
Very slow		5	0.7		13	1.8	<0.001^b^
Slow		54	7.3		104	14.2	
Average		315	42.5		408	55.7	
Fast		316	42.6		183	25.0	
Very fast		51	6.9		24	3.3	
Education level ^f^							
Low		111	15.0		122	16.7	0.58^b^
Medium		217	29.3		219	29.9	
High		413	55.7		391	53.4	
Smoking status							
Non-smoker		664	89.6		676	92.3	0.07^b^
Smoker		77	10.4		56	7.7	

^a^Independent samples T-test

^b^Chi-square test

^c^Suspected under reporters (i.e., reported energy intake/calculated basal metabolic rate < 1.35) excluded

^d^BMI ≥25 kg/m^2^

^e^BMI ≥30 kg/m^2^

^f^Education level: low (i.e., no education, primary education, lower or preparatory vocational education, or lower general secondary education), medium (i.e., intermediate vocational education or apprenticeship, or higher general secondary education or pre-university secondary education) and high (i.e., higher vocational education or university)

Table 1 shows the prevalence of all five SRER-categories in men and women. The SRER-categories were distributed differently for men and women (chi-square, *P* < 0.001); compared to women, men more often reported to be fast eaters. Furthermore, 931 participants (men/women = 458/473) answered the eating rate question twice (Table 2). A κ-value of 0.64 was found for the level of agreement between the answers to both questions.

**Table 2:** Frequency of self-reported eating rate-categories (SRER) in participants that answered the eating rate question twice^a^

		SRER second time				
		Very slow	Slow	Average	Fast	Very fast
SRER first time	Very slow	13	3	0	0	0
	Slow	5	87	23	2	0
	Average	0	36	367	58	0
	Fast	1	0	49	226	18
	Very fast	0	0	2	18	23

^a^κ-value = 0.64

Tables 3 and 4 show the characteristics of the participants by SRER-category (i.e., slow, average, fast). A positive linear association was found between BMI and SRER-category in both men (*r* = .08, *P* = 0.03) and women (*r* = .16, *P* < 0.001). Also waist circumference and body fat percentage showed a positive association with SRER in women (waist circumference *r* = .10, *P* < 0.01; body fat percentage *r* = .14, *P* < 0.001), but not in men (waist circumference *r* = .01, *P* = 0.74; body fat percentage *r* = .01, *P* = 0.71). In addition SRER was positively associated with moderate-to-vigorous activity, restrained eating and external eating in men, and positively associated with emotional, restrained and external eating in women.

**Table 3:** Characteristics (mean ± SD) of the participants by self-reported eating rate-category, within the total population and in men and women separately

	Self-reported eating rate									*P* ANOVA	*P* linear trend
	Slow			Average			Fast				
	n	mean	SD	n	mean	SD	n	mean	SD		
Total											
Age (yrs.)	176	53.0	13.1	723	55.1	11.0	574	54.6	12.1	0.11	.42
BMI (kg/m^2^)	176	24.8	4.1	723	25.5	4.0	574	26.6	3.9	<0.001	<0.001
Waist circumference (cm)	175	88.2	13.1	721	90.4	12.3	572	93.6	12.2	<0.001	<0.001
Body fat percentage (%)	145	29.9	9.0	647	30.2	8.6	504	28.6	8.8	0.01	0.01
Energy intake (MJ/day)	158	8.4	2.3	662	8.5	2.4	525	8.9	2.7	<0.01	<0.01
Energy intake (MJ/day) ^a^	75	9.9	1.8	300	10.2	2.0	209	11.3	2.4	<0.001	<0.001
Emotional eating	176	2.13	0.69	723	2.06	0.71	574	2.17	0.73	0.02	0.09
Restrained eating	176	2.86	0.79	723	3.01	0.72	574	3.03	0.69	0.03	0.03
External eating	176	2.61	0.48	723	2.67	0.42	574	2.78	0.43	<0.001	<0.001
Moderate-to-vigorous activity (h/day)	164	35.4	19.5	663	33.8	17.0	535	35.4	18.0	0.24	0.53
Sedentary activity (h/day)	164	37.6	35.0	663	35.5	29.0	534	37.4	24.4	0.45	0.68
Men											
Age (yrs.)	59	57.9	10.7	315	58.2	10.0	367	56.7	11.1	0.16	0.10
BMI (kg/m^2^)	59	25.7	3.9	315	26.3	3.4	367	26.7	3.5	0.09	0.03
Waist circumference (cm)	58	96.1	10.9	314	97.0	10.7	365	96.9	10.7	0.82	0.74
Body fat percentage (%)	52	23.8	6.9	294	24.9	6.8	337	24.7	6.4	0.56	0.71
Energy intake (MJ/day)	54	10.0	2.4	295	9.3	2.5	342	9.5	2.8	0.25	0.89
Energy intake (MJ/day) ^a^	27	11.5	1.6	125	11.4	2.0	136	12.0	2.2	0.04	0.03
Emotional eating	59	2.01	0.68	315	1.85	0.61	367	2.01	0.67	<0.01	0.053
Restrained eating	59	2.65	0.79	315	2.84	0.75	367	2.92	0.69	0.02	<0.01
External eating	59	2.62	0.48	315	2.64	0.42	367	2.76	0.43	0.001	<0.001
Moderate-to-vigorous activity (h/day)	57	31.5	19.0	298	32.6	17.8	341	35.4	18.9	0.10	0.04
Sedentary activity (h/day)	57	37.6	19.4	298	35.0	18.8	341	37.8	22.9	0.23	0.30
Women											
Age (yrs.)	117	50.6	13.5	408	52.7	11.1	207	50.8	12.9	0.09	0.75
BMI (kg/m^2^)	117	24.4	4.2	408	25.0	4.3	207	26.4	4.6	<0.001	<0.001
Waist circumference (cm)	117	84.2	12.4	407	85.3	10.9	207	87.6	12.3	0.02	<0.01
Body fat percentage (%)	93	33.2	8.3	353	34.6	7.4	167	36.5	7.7	<0.01	<0.001
Energy intake (MJ/day)	104	7.6	1.8	367	7.8	2.0	183	7.8	2.3	0.58	0.39
Energy intake (MJ/day) ^a^	48	9.0	1.1	175	9.4	1.5	73	9.8	1.9	0.02	<0.01
Emotional eating	117	2.19	0.69	408	2.22	0.73	207	2.45	0.74	0.001	<0.001
Restrained eating	117	2.97	0.77	408	3.4	0.66	207	3.21	0.65	<0.01	<0.001
External eating	117	2.60	0.48	408	2.69	0.43	207	2.82	0.44	<0.001	<0.001
Moderate-to-vigorous activity (h/day)	107	37.5	19.5	365	34.7	16.4	194	35.3	16.4	0.31	0.41
Sedentary activity (h/day)	107	37.7	41.1	365	35.9	35.2	193	36.6	27.0	0.90	0.87

^a^Suspected under reporters (i.e., reported energy intake/calculated basal metabolic rate < 1.35) excluded

**Table 4:** Frequency of participant characteristics by self-reported eating rate-category, within the total population and in men and women separately

	Self-reported eating rate						*P* chi-square test
	Slow		Average		Fast		
	n	%	n	%	n	%	
Total^a^							
Prevalence of overweight^d^	75	42.6	354	49.0	353	61.5	<0.01
Prevalence of obesity^e^	18	10.2	89	12.3	102	17.8	<0.01
Prevalence of smoking	19	10.8	56	7.7	58	10.1	0.23
Education level^f^							
Low	29	16.5	126	17.4	78	13.6	0.19
Medium	44	25.0	220	30.4	172	30.0	
High	103	58.5	377	52.1	324	56.4	
Men^b^							
Prevalence of overweight^d^	29	49.2	196	62.2	235	64.0	0.09
Prevalence of obesity^e^	6	10.2	48	15.2	64	17.4	0.33
Prevalence of smoking	6	10.2	31	9.8	40	10.9	0.71
Education level^f^							
Low	12	20.3	52	16.5	47	12.8	0.37
Medium	13	22.0	92	29.2	112	30.5	
High	34	57.6	171	54.3	208	56.7	
Women^c^							
Prevalence of overweight^d^	46	39.3	158	38.7	118	57.0	<0.001
Prevalence of obesity^e^	12	10.3	41	10.0	38	18.4	0.01
Prevalence of smoking	13	11.1	25	6.1	18	8.7	0.16
Education level^f^							
Low	17	14.5	74	18.1	31	15.0	0.46
Medium	31	26.5	128	31.4	60	29.0	
High	69	59.0	206	50.5	116	56.0	

^a^Slow, *n* = 176; Average, *n* = 723; Fast, *n* = 574

^b^Slow, *n* = 59; Average, *n* = 315; Fast, *n* = 367

^c^Slow, *n* = 117; Average, *n* = 408; Fast, *n* = 207

^d^BMI ≥25 kg/m^2^

^e^BMI ≥30 kg/m^2^

^f^Education level: low (i.e., no education, primary education, lower or preparatory vocational education, or lower general secondary education), medium (i.e., intermediate vocational education or apprenticeship, or higher general secondary education or pre-university secondary education) and high (i.e., higher vocational education or university)

In both men and women SRER was not associated with energy intake before excluding participants suspected of under reporting energy intake (Table 3). In total, 754 participants (men/women = 399/355) were identified as underreporting their energy intake. After excluding these participants SRER was positively associated with energy intake in men (*r* = .13, *P* = 0.03 (*n* = 296)) and women (*r* = .17, *P* < 0.01 (*n* = 288)). In turn, energy intake was positively associated with BMI in men and women after excluding suspected under reporters and adjusting for sedentary and moderate-to-vigorous activity; regression coefficients were 0.28 kg/m^2^ /MJ (95% CI: 0.12, 0.44) for men and 0.55 kg/m^2^/MJ (95% CI: 0.30, 0.79) for women (men/wome*n* = 273/281).

Model 3 in Table 5 shows the associations between SRER-categories and BMI after adjusting for age, smoking, education level, emotional eating, restrained eating, and external eating. BMI was 1.13 kg/m^2^ higher in self-reported fast-eating women compared to self-reported average-speed-eating women. The BMI of self-reported slow-eating women was not significantly different from that of self-reported average-speed-eating women. In men the BMI of both self-reported slow- and fast-eaters was not significantly different from that of self-reported average-speed-eaters. The relation between SRER and BMI was not significantly different between men and women (interaction effect in multiple linear regression: *P* = 0.06). Furthermore, when energy intake, moderate-to-vigorous activity and sedentary activity were added to the main model, the results remained similar (men/women = 273/281) (Table 5, Model X).

**Table 5:** Association between self-reported eating rate (SRER) and BMI within the total population and in men and women separately according to multiple linear regression analysis and linear trend analyses

Independent variables	Model 1^a^		Model 2^b^		Model 3^c^		Model X^d^	
	Partial regression coefficient	95% CI or *P*	Partial regression coefficient	95% CI or *P*	Partial regression coefficient	95% CI or *P*	Partial regression coefficient	95% CI or *P*
Total		(*n* = 1473)		(n = 1473)		(n = 1473)		(*n* = 554)
SRER-categories								
Slow	−0.69	(−1.35, −0.03)	−0.57	(−1.22, 0.08)	−0.48	(−1.11, 0.15)	−0.47	(−1.20, 0.27)
Average	0.00	(reference)	0.00	(reference)	0.00	(reference)	0.00	(reference)
Fast	1.03	(0.59, 1.47)	1.09	(0.66, 1.52)	0.90	(0.48, 1.32)	0.58	(0.05, 1.10)
Linear trend		<0.001		<0.001		<0.001		<0.01
Men		(n = 741)		(n = 741)		(n = 741)		(n = 273)
SRER-categories								
Slow	−0.57	(−1.55, 0.41)	−0.56	(−1.51, 0.40)	−0.47	(−1.40, 0.47)	−0.42	(−1.49, 0.65)
Average	0.00	(reference)	0.00	(reference)	0.00	(reference)	0.00	(reference)
Fast	0.40	(−0.14, 0.93)	0.51	(−0.001, 1.03)	0.29	(−0.22, 0.80)	0.28	(−0.38, 0.94)
Linear trend		0.03		<0.01		0.08		0.18
Women		(*n* = 732)		(n = 732)		(n = 732)		(*n* = 281)
SRER-categories								
Slow	−0.56	(−1.46, 0.34)	−0.44	(−1.34, 0.45)	−0.24	(−1.11, 0.63)	−0.34	(−1.36, 0.67)
Average	0.00	(reference)	0.00	(reference)	0.00	(reference)	0.00	(reference)
Fast	1.40	(0.67, 2.14)	1.51	(0.78, 2.23)	1.13	(0.43, 1.84)	0.71	(−0.15, 1.56)
Linear trend		<0.001		<0.001		<0.01		0.06

^a^Crude model

^b^Crude model with age, smoking and level of education

^c^Crude model with age, smoking, level of education, emotional eating, restrained eating and external eating

^d^ Crude model with age, smoking, level of education, emotional eating, restrained eating, external eating, energy intake, moderate-to-vigorous activity and sedentary activity (excl. Suspected under reporters of energy intake; i.e., reported energy intake/calculated basal metabolic rate < 1.35)

Finally, self-reported fast eaters were at higher risk to be overweight (i.e., BMI ≥ 25 kg/m^2^) compared to the other participants (i.e., self-reported average- plus slow-speed eaters) with an adjusted odds ratio of 1.73 (95% CI: 1.38, 2.17). Within women this adjusted odds ratio was 2.05 (95% CI: 1.44, 2.91), while within men this was 1.13 (95% CI: 0.82, 1.56). These odds ratios were not significantly different for men and women (interaction effect in logistic regression: *P* = 0.09).

## Discussion

In these studies self-reported eating rate was validated against actual eating rate, and the association between self-reported eating rate (SRER) and weight status was investigated in a Dutch population. The validation study confirmed that self-reported eating rate was positively associated with actual eating rate. The cross-sectional data from the NQplus cohort showed that self-reported eating rate was positively associated with BMI among both men and women. After adjusting for confounders self-reported eating rate remained significantly associated with BMI in women; fast eaters had on average a 1.13 kg/m^2^ higher BMI compared to average-speed eaters. In men this relation was no longer significant after adjusting for confounders; nonetheless, the direction of the association was still in the expected direction. Overall, self-reported fast eaters were more likely to be overweight compared to self-reported non-fast eaters.

These findings are in line with previous studies investigating the association between SRER and weight status. In the current study the adjusted odds ratio for being overweight, comparing self-reported fast eaters to non-fast eaters, was 1.73 (95% CI: 1.38, 2.17); where Ohkuma et al. [7] found a pooled odds ratio of 2.15 (95% CI, 1.84–2.51) in their meta-analysis. This shows that previous findings from Asian populations may translate to non-Asian populations. The current study was the first to investigate this association in a non-Asian population that included men and objectively measured height, weight, waist circumference and body fat percentage.

Eating rate is expected to affect weight status via energy intake. If people eat fast, calories pass through the oral cavity quickly, are not sensed and do not bring about an adequate satiety response, resulting in an increased intake [32–36]. The current findings are in line with this. After excluding suspected under reporters, energy intake was positively associated with BMI. More importantly, energy intake was positively associated with SRER. Previous studies also found positive relations between energy intake and SRER, although not always statistically significant. More accurate measurements of energy intake might reveal stronger relations between energy intake, SRER and BMI. The problem with dietary assessment methods is that the measurement error depends on BMI; overweight people are more likely to underreport energy intake [24]. Excluding under-reporters does not completely resolve this issue.

Furthermore, the validation study confirms that on a group level self-reported eating rate reflects actual eating rate in young adults. Actual eating rate increased proportionally with SRER-categories, and like Petty et al. [18], we found that actual eating rate was significantly higher in self-reported fast eaters compared to self-reported slow and average-speed eaters. We assumed that these findings will also translate to older adults, as eating rate appears to be a stable personal characteristic [3–5]. Moreover, we did not find an association between SRER and age in the cross-sectional study.

However, when examining the results of the validation study at the individual level, only half of the participants correctly classified themselves according to their actual eating rate. The kappa-values showed that after correcting for chance the remaining agreement between SRER and tertiles of actual eating rate was only 25%, which is considered ‘fair’ [28]. As such, SRER might not be a good measure for actual eating rate at the individual level. In the cross-sectional study, however, SRER was used as a measure of eating rate on a group-level. Furthermore, this imperfect agreement between SRER and actual eating rate might mean that the results of the cross-sectional analysis underestimate the true association between eating rate and BMI.

Different explanations exist for the agreement between SRER and actual eating rate being only fair. First, people might not be aware of their eating rate, although this does not seem to be the case. There is good agreement between the answers of people that answered the eating rate question twice, which shows that they have a fixed image of their eating rate. Second, people might interpret eating rate differently than scientists. Third, people are limited to their own observations to evaluate their eating rate and that of others. People do not monitor their eating rate like scientists would: i.e., using a stopwatch and kitchen scale. So how do they answer the eating rate-question? They, for example, could base their answer on how long it takes them to finish one portion or the length of their meals. Finally, they could use different people as a reference.

More intervention studies are needed to investigate if there is a causal relation between (self-reported) eating rate and BMI, and whether this is mediated by long-term energy intake. Based on evidence from experimental studies, these intervention studies should focus on increasing oral sensory exposure time. Some interventions targeting eating rate have already been examined. Spiegel et al. [1] included advice on reducing eating rate in a weight loss program. Participants successfully reduced eating rate, which resulted in weight loss. However, the slower eating rate was not maintained over time. McGee et al. [8] performed a four-month intervention with an ‘oral volume restriction device’. This device was worn in the upper palate during a meal, which reduced bite size and thereby eating rate. Participants that used the device most lost more weight. Further advancements could be made by using new technologies, which offer useful tools for both monitoring and altering eating rate. The SPLENDID-system and 10SFork constitute examples of such new technologies [37, 38]. Both provide real-time feedback on eating rate. Usage of such technologies seems to be the logical next step for future research.

## Conclusions

The two current studies showed that 1) self-reported eating rate reflects actual eating rate on a group-level, but not at the individual level, and 2) that self-reported fast eating is associated with a higher BMI in a Dutch, adult population, although this association was more pronounced in women. Lowering eating rate might be a promising strategy in tackling obesity. However, first more empirical evidence is needed to confirm the causal relationship between (self-reported) eating rate and BMI, and to show the effectiveness of interventions targeting eating rate.

## Abbreviations

AQUAA: Activity questionnaire for adults and adolescents
BMI: Body mass index
CI: Confidence interval
DEBQ: Dutch eating behavior questionnaire
SQUASH: Short questionnaire to assess health-enhancing physical activity
SRER: Self-reported eating rate
